# Automated Monitoring and Clinical Notifications of Patient State for Neuropsychiatric Neuromodulation Studies

**DOI:** 10.1109/EMBC58623.2025.11252732

**Published:** 2025-07

**Authors:** Tomasz M. Fraczek, Yewen Zhou, Thomas P. Kutcher, Raphael A. Bechtold, Saipravallika Chamarthi, Nora Vanegas Arroyave, Wayne K. Goodman, Sameer A. Sheth, Jeffrey A. Herron, Nicole R. Provenza

**Affiliations:** Baylor College of medicine, Houston, TX 77024, USA; Baylor College of medicine, Houston, TX 77024, USA; Rice University, Houston TX, 77005, USA; University of Washington Seattle WA, 98195, USA; Baylor College of medicine, Houston, TX 77024, USA; Baylor College of medicine, Houston, TX 77024, USA; Baylor College of medicine, Houston, TX 77024, USA; Baylor College of medicine, Houston, TX 77024, USA; University of Washington Seattle WA, 98195, USA; Baylor College of medicine, Houston, TX 77024, USA; Rice University, Houston TX, 77005, USA

## Abstract

The proliferation of wearable technologies and the internet of things has revolutionized clinical research by opening the door to continuous at-home monitoring. These systems give clinicians better insight into patient well-being throughout daily life. Concurrently, new deep brain stimulation (DBS) devices have opened avenues for continuous neural recordings in the background of everyday activities. Behavioral data passively collected from wearables could lead to new insights into the neural mechanism underlying the pathophysiology of neurological or psychiatric disorders. Additionally, reliable multi-device monitoring systems could be used to detect or predict symptoms of a disorder or side effects of an intervention. This is particularly important in investigational neuromodulation trials in which stimulation parameters can be adjusted in the clinic to manage symptoms and side effects but the chronic response of the patient’s symptoms to stimulation out of the clinic is still under investigation. Here we demonstrate a prototype continuous monitoring and email-based warning notification system, designed to incorporate data from multiple wearable devices and detect changes in clinical status for patients with neuropsychiatric disorders enrolled in DBS studies. The system is built with custom Python packages that collect data from multiple third-party APIs and synchronize different data modalities to a common second-resolution database. This backend enables automated analysis and visualization, supporting both real-time monitoring and retrospective review of patients undergoing deep brain stimulation treatment., It has been tested and deployed to the real world with several patients who wore Oura rings while undergoing deep brain stimulation treatment for obsessive-compulsive disorder. The system has been able to reliably detect changes in measurable changes in the individual’s behavior and has operated successfully for over 8 months.

## I. Introduction

Modern clinical neuroscience is increasingly leaving the clinic and focusing more and more on at-home monitoring of the patient [1-7]. This has the potential advantage of helping clinicians monitor patient well-being throughout daily life and ensure that the measured metrics reflect individuals’ daily functioning of the patient [4, 8]. This is particularly important for treatments such as deep brain stimulation (DBS), where the level of stimulation often needs to be adjusted, and these adjustments can quickly result in clinically meaningful changes rapidly and significantly affecting the patient [9-11]. Already in the field of DBS for Parkinsons’ disease (PD), at-home monitoring of patient state using wearable devices has demonstrated great improvements in patient well-being, especially in terms of motor outcomes [1, 6, 12]. This success has led to the development and successful deployment of large monitoring applications for DBS patients with PD [1, 13, 14]. Some of these have already been deployed as an enterprise-scale remote monitoring and digital health platform in clinical studies of movement disorders [13]. However, existing efforts have focused on motor symptoms and are not available as open-source software that can be customized.

In the field of psychiatric DBS, the need for robust at-home monitoring of the patients is arguably even more pressing [15-17]. DBS for obsessive-compulsive disorder (OCD), approved under a humanitarian device exemption by the US FDA, significantly improves symptoms in 66% of treatment-resistant patients [1, 2]. DBS has shown varying degrees of success in various other psychiatric disorders, including depression [20-22], Tourette syndrome [3], substance use disorders [4, 5], and eating disorders [6]. However, DBS programming for both neurological and psychiatric disorders requires multiple visits to the clinic for optimization over a period of several months to a year [6, 9, 14]. One common side effect of overstimulation across disorders is overly disinhibited behavior, associated with reduced need for sleep, impulsivity, elation, and/or anxiety [10, 11, 19, 27-29]. We carefully monitor the emergence of symptoms related to disinhibition in the clinic immediately following programming visits. If related symptoms are detected in the clinic (e.g., increased talkativeness, increased motor activity, overly social behavior), stimulation can be reduced to avoid onset of disinhibited behavior. However, these symptoms often emerge over the course of several hours to days after the patient leaves the clinic [4, 19]. In these cases, continuous passive and robust monitoring of the clinical status, tuned specifically to the expected changes for the chosen disorder and stimulation target could lower the occurrence of adverse events by allowing the clinical team to reach out to the patient and adjust stimulation as necessary.

In our upcoming clinical study of DBS for treatment resistant bipolar depression (TRBD; NCT06599099), such monitoring is required as a critical element of the patient safety infrastructure. This is because one of the main concerns for TRBD DBS is the induction of dangerous mixed manic-depressive states [29, 30]. Past work has identified dramatically reduced sleep and increased levels of activity as signs of hypomania/mania [7]. These metrics can be captured by modern wearable devices, such as the Oura ring and Apple Watch [4, 5, 7, 31], and could be monitored automatically to be able to detect such dangerous changes in the patient state, allowing the clinical team to intervene to improve outcomes and prevent adverse events.

We have developed a prototype of such a monitoring and clinician notification system. Here we present results demonstrating this system both in a controlled engineering verification effort, and in a preliminary deployment with three patients implanted with DBS for OCD. Through the early testing of this system, we demonstrate the capacity to track sleep and activity levels, two measures that have previously shown promise for tracking symptoms relevant to psychiatric disorders [5, 4, 31]. Furthermore, we discuss plans for further customization of this system to support an upcoming clinical trial evaluating safety and efficacy of DBS for TRBD, as part of the critical risk-mitigation infrastructure for our study.

## II. System Overview

**Figure 1:**
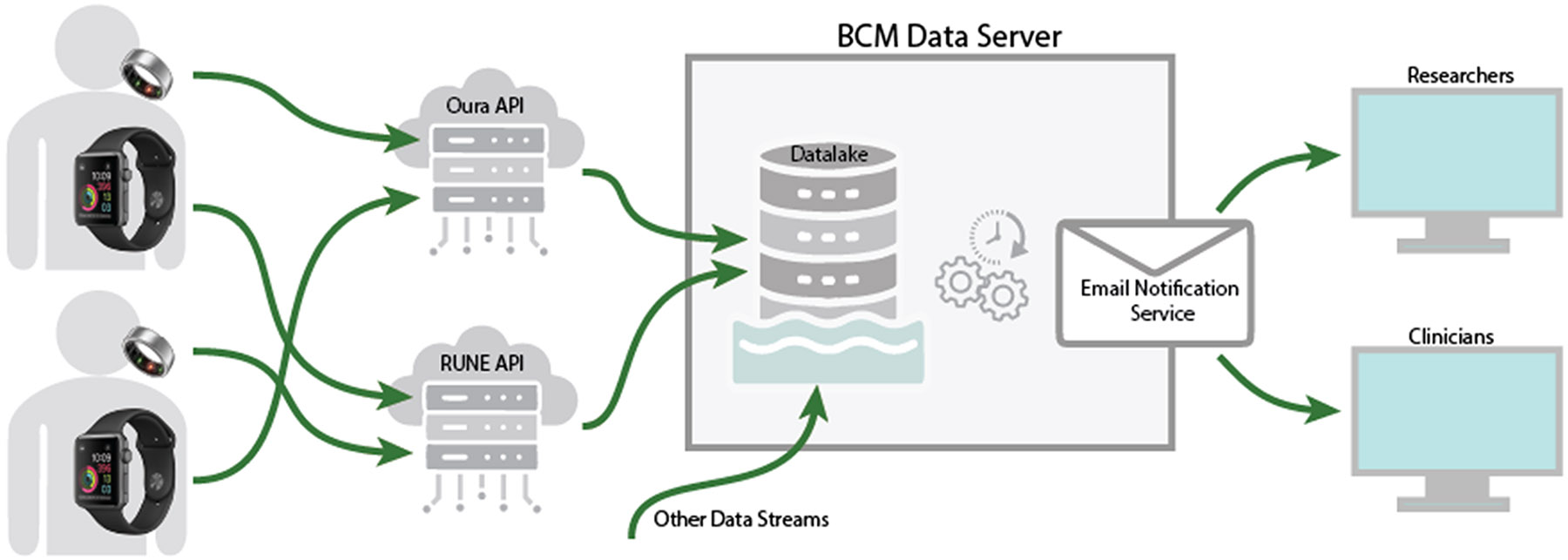
Block diagram of the data acquisition and email notification service. Data is acquired by each wearable device and synchronized independently to each device manufacturers respective servers where it is made available via the API. From there, our automated systems automatically download new data to the data lake hosted on our main processing server. A daily email service processes the data, extracts any requested metrics, and looks for triggers on any of the configured warnings. An email summarizing these results is then sent to all subscribed research and clinical staff.

The system, shown in Figure 1, is constructed from a series of sequential processing elements, which start with the patient syncing the data from their Oura ring to their phone by opening the Oura app, so it can be uploaded to the Oura data servers. To limit the burden on the patient and help with compliance, we only require the patient to perform this synchronization step once a day, in the late morning. This time helps ensure that the uploaded data fully captures the patient’s sleep pattern from the previous night, allowing us to monitor daily sleep, reliably and with minimal delay.

**Figure 2:**
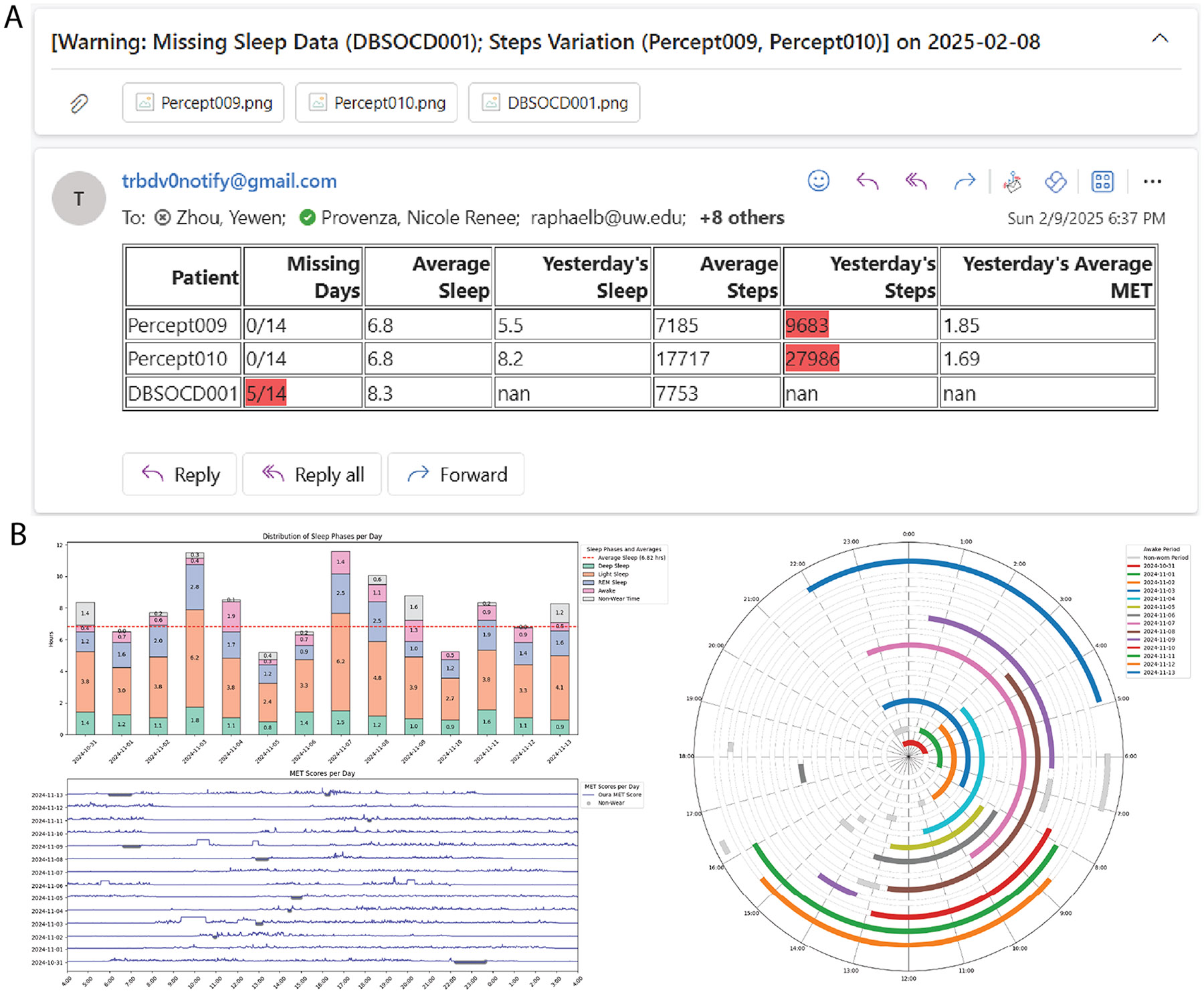
Example of the daily notification emails. (A) Screenshot of one of the most recent email notifications from our real-world deployment, showing the clearly labeled warnings in the email header, as well as highlighting of the relevant cells in the patient summary statistics table. Detailed plots of the data are included in the email attachments. (B) shows the sleep detail plot from the email which triggered a patient check in, because of a sudden shift in the sleep schedule.

Shortly after the scheduled upload of the data, an automated process retrieves the new data from the Oura API and saves it to our central data storage location where it is then ingested by our python processing code. This code extracts all the data from the .json files saved for each modality and each day. The data is then assembled into a continuous stream to best evaluate several clinically meaningful metrics. For our deployments, we selected metrics including total sleep duration, sleep phases, and activity level, as past research has shown these features as promising indicators of hypomania/mania [7, 10, 11, 31-33]. We defined our detection threshold for behavioral changes as a 25% change from baseline for any of these metrics. If a 25% change is detected, a warning is triggered. In addition, if the total non-wear time for a day is over 6 hours a warning is triggered, as the reliability of all metrics decreases as non-wear time increases. A summary of this analysis is the sent out via email, using python’s smtplib. An example of this email is shown in Figure 2. If no warnings were triggered, then the email is titled [All Clear]. If any warning was triggered, then the email contains the header [Warning], along with the name of the warning and the patient(s) for whom the warnings were triggered. This allows clinicians and research staff to know immediately when opening their inbox whether there is a cause for concern that should be further investigated.

The body of an email contains a table of the summary statistics computed for each patient, as shown in Figure 2A. For every warning that was triggered, the relevant cells in the table are highlighted red, allowing users to quickly narrow in on the problem area. To help with further evaluation, a collection of detailed plots, created using a custom python matplotlib plotting class, for each patient is included in the attachments. Based on this information, the clinical team can determine if the patient should be contacted for further inquiry and potential changes to stimulation parameters.

The system is also designed to help monitor compliance. The table of summary data displays the number of recent days for which there is not sufficient data available to compute the desired metrics. This may indicate that either the device was not on for a significant portion of the day, or that the patient has not yet uploaded some of their most recent data. Since reliable data acquisition is required for the system to ensure patient safety, the email will contain a warning whenever a patient’s most recent data (i.e., data collected over the previous 24 hours) is not available. The clinical team can then reach out to the patient to encourage compliance and gauge clinical status.

We verified the ability of our system to capture meaningful changes in clinical metrics, as defined by a panel of expert clinicians, in a controlled environment. Two lab members wore an Oura ring and an Apple smart watch for a period of several weeks, while keeping a diary of their sleep, socialization, and overall activity levels. The diary was created to consistently log behavior, ensuring cohesive and uniform notes across all volunteers. To achieve this, we developed a structured log that captured four key aspects: sleep, exercise, social interaction, and general location. For sleep, start and end times were recorded to establish a ground truth for verifying device-based sleep monitoring and ensuring sleep duration accuracy. Exercise logs included time, type, and intensity to assess whether wearables could detect overall changes in exercise levels. Social interactions and general location (logged as either home or work) to use in future verification of in-development socialization and interaction features from audio recordings and GPS positioning respectively. The primary objective of this diary was to establish a reliable behavioral ground truth that could be used to validate the wearables’ ability to accurately detect specific changes in patient behavior.

The system has also been deployed in a real-world clinical environment. As part of an ongoing clinical study of DBS for OCD (NCT05915741), patients have been provided with Oura rings to help track their symptoms over time and help evaluate effectiveness of the DBS stimulation on their symptoms. Data for three patients, with compliance records >= 50% (wore their Oura ring on at least half of the days), has been included in a trial deployment of our monitoring system. The notification service has been continuously deployed for over eight months, while receiving constant updates and improvements to software reliability and visualizations.

## III. Results

**Figure 3:**
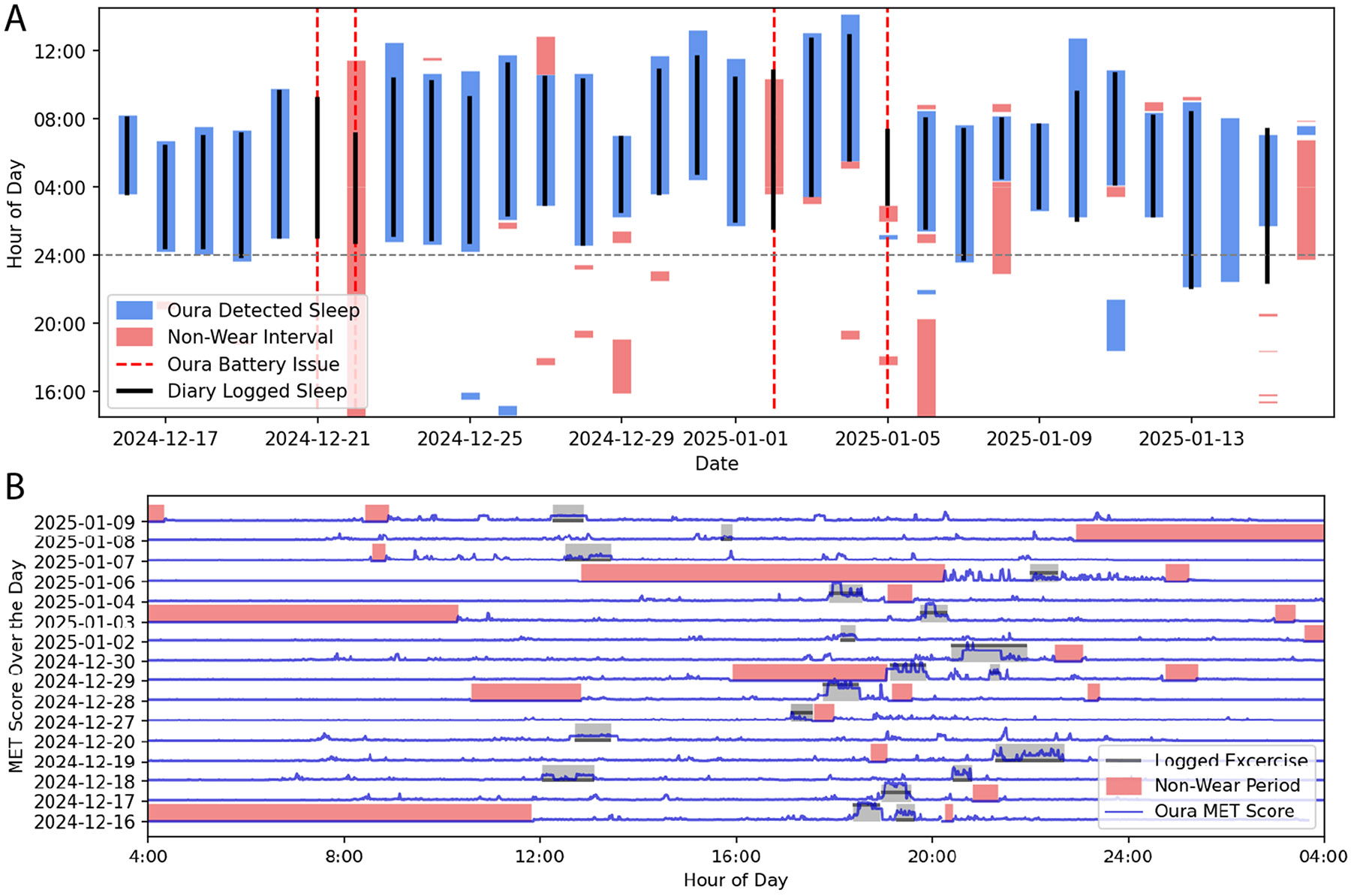
Verification Testing Results: Demonstration of the agreement between metrics computed from the Oura ring and diaries kept by one of our lab members for both sleep timing and exercise intervals, during a controlled engineering test period. In the upper plot, the bedtime periods detected by the Oura ring, shown in blue, and tracked in a sleep diary by a research volunteer, shown as the black lines. The main reason for disagreements between the two sets of sleep data, are compliance issues, shown in red, both not wearing the ring, and the ring running out of charge while worn. In the bottom plot, activity (metabolic equivalent time, MET) scores from the Oura ring over the course of the day are shown in blue and logged exercise periods are shown with grey highlighted areas. The height of the dark grey line indicates the logged exercise intensity, on a 3 point, low-medium-high scale.

Our system has demonstrated an ability to collect wearable signals, process the data, and notify of behavioral changes that may indicate an unintended change in clinical status. Based on conversations with a panel of clinical experts, we determined that a 25% change in daily sleep duration and a 25% change in average daily activity would serve as primary metrics for monitoring patient safety. Our ability to detect these changes was tested in a controlled environment. Plots comparing the objective, wearable-based measures of a volunteer lab member to the logged events in their diaries are shown in Figure 3. Overall, we see that the bedtime periods detected by the Oura ring track well with the sleep periods logged in our researcher volunteer’s sleep diary (Intersection over Union of 0.74 with compliance dates excluded). The detected periods of heightened activity match with exercise periods logged by the volunteer. The most common cause of inconsistencies between the bedtime periods logged by our team member and those detected by the Oura ring were device disconnects, primarily as a result of the battery running out partway through the night. The occurrence of these, even with healthy, trained engineers aware of the need for reliable data highlights how easy it can be to forget to charge the device. However, with assistance from the clinical team, we hope that we can help ensure that participants regularly wear and charge the Oura ring. The reliable match between the logged values and the detected measures suggest that our current monitoring devices would be able to meet the 25% change threshold required by our clinical partners.

An example of the notification email from the test deployment to the cohort of three OCD patients and its attachments are shown in Figure 2A. Already using this system, we have been able to detect and respond to significant changes in patient state. In one particularly prominent example, a warning was triggered for one of our patients having very little sleep. Further investigation of the detail plots attached in the email, shown in Figure 2B, showed that the patient had been sleeping during the day instead of at night. This prompted an urgent check-in with the patient. Upon checking in, the clinical team learned that the patient had taken a night shift for their job and their sleep schedule had changed. Although this case did not result in a clinical intervention or change in therapy, it did demonstrate the ability of the notification service to detect and alert to sudden changes in patient state and allow the team to take immediate action to ensure patient well-being.

## IV. Future Developments

The experience gained with the development, deployment, and usage of this prototype system will help guide development of future iterations. Overall, we believe that the prototype has already demonstrated much of the required capabilities in ensuring patient safety. We have demonstrated that the system has successfully been able to detect behavioral changes that may indicate an unintended change in clinical status, both in an engineering trial dataset and during a real-world deployment to a cohort of OCD patients. Given these capabilities, we think that this system shows promise for facilitating data-driven strategies for delivering neuromodulation therapy.

There are several areas where further development is required in order for this prototype system to realize its full potential. First, the basic science of using wearable sensors to model patient well-being in terms of behavior relevant to bipolar disorder (i.e. mood and energy swings) or other psychiatric disorders is still under investigation. However, through the extensive collection of data, we hope to create more sophisticated behavioral models than simple sleep duration or activity levels to not only extract patient state in a more accurate manner, but also in a more time responsive and sensitive manner. As such, future versions of the monitoring system will need to support complex multi-dimensional models of patient state that integrate multiple types of data from multiple devices. Such models will allow much higher sensitivity and will hopefully allow the warnings to be triggered off a more accurate representation of the patient well-being instead of the proxy metrics used here. Sensing-enabled DBS implants will further allow for the integration of data collected from wearables with neural data from the implanted device to develop and validate neural biomarkers of disease state, as well as building models to predict current and future biomarker estimates from the wearable device measures. Tuning all these models, however, will require the collection of training data from patients, combined with labels of patients’ state, which will only be possible partway through the study. During the initial phase of the study, close, in person, monitoring at specially instrumented, apartment-like suite will ensure patient safety over the first 24-hour period after stimulation therapy is activated. Once the patient goes home, initial simple heuristics like those demonstrated here will help the clinical team track the patient. Moreover, as more complex models can be more prone to failures, the current metrics will continue to serve a valuable role as a safety fallback.

One core limitation of the current system is related to data security. Although the list of email addresses that the notifications are sent to is controlled, and only includes people authorized to view patient data, email itself is in general not a secure way to share data. This limits the current version of the email notification system to only include non-PHI (patient health information) data, and all data must be anonymized. This in turn limits the complexity and detail that can be provided in this email system. Future versions of our full system will include a web dashboard which will require secure login. This will allow us to display more detailed patient-specific data across a wider variety of data modalities and more sophisticated model outputs. Clinicians will then receive simplified, non-PHI summary emails with notifications and links to check patient details on our web portal.

Device issues were one of the main causes of inaccuracy in the monitoring and notification system. As the complexity of monitoring systems and the number of possible devices used in neuropsychiatric neuromodulation increases, the potential for these errors will grow substantially. As seen in the test deployment, keeping one device charged can be challenging, leading to compliance issues, data loss, and potential failures to detect changes in patient state. We expect that patients may have trouble keeping multiple devices always charged, requiring limitations on the number of wearables that can be practically deployed. Moreover, as any significant loss of data, either due to non-wear periods or battery failure, will need to trigger a warning, and may require the clinical team to intervene. Similarly, the reliance on third-party APIs such as Rune Labs and Oura could lead to critical failures or monitoring problems in the event of API changes or service discontinuation. Certain devices, such as the implanted DBS system, cannot synchronize data remotely, and instead require an in-person interaction with the patient to download the data. This means that this data cannot be used as part of the automated monitoring infrastructure. Moreover, as new devices become available, synchronization of data from multiple distributed sources will need to be verified. In all these cases, a robust well-designed data infrastructure will help ensure that patient monitoring continues.

One aspect of the system that will require particular care through the development process is the sensitivity of the system, and the balance of false positives and false negatives. False negatives are of course dangerous, as they could lead to oversight of a dangerous change in patient state. However, an excess of false positives could also be problematic, as they could drown out the real warnings signs and lead human operators to reduce their trust in the validity of the warnings given by the warning system, known as the cry-wolf effect [34, 35]. Tuning the warnings will likely need to be a continued iterative process. Starting and the best estimate of a reasonable warning threshold by expert clinicians, the warning thresholds will need to be continually adjusted to the level that will maximize patient safety. As this is not possible to do in general, one potential solution may be to provide the clinical safety team with the ability to adjust trigger thresholds in the deployed application.

Deployments for different cohorts of patients with other conditions and undergoing varied treatments will also likely require specially chosen metrics. Although sleep is important for health in all populations, mania and hypomania are concerns primarily in the depression and TRBD populations. In contrast, monitoring for a cohort of PD patients would focus more on motor function measures including changes in mobility during sleep phases, as demonstrated by multiple studies in this field [2, 12-14]. Different measures may require data from a variety of sensors on a variety of devices, meaning that our system must be developed to handle this level of flexibility and customizability. For psychiatric DBS in particular, this tuning process may need to be highly patient specific, as demonstrated by the case of our night-shift patient. In many patients, a sudden shift of sleep schedule could be indicative of mania but in a patient with a highly variable work schedule, warnings like these would almost always be false positives.

Feedback from our clinical partners to the deployed monitoring and email notification system has been overwhelmingly positive. They noted that regular notifications, in combination with customizable metrics, helped them keep a better eye on their patients. As a result of this trial deployment with 3 OCD patients, our clinician partners have asked us to keep the notifications on, and to expand the system to their full patient cohort. Continued development of our monitoring platform will include a researcher-focused scripting client and a clinician-focused web dashboard. We hope these tools will allow the research team to rapidly iterate and design novel and more accurate measures of patient state, and help the clinical team more reliably monitor the patients in their clinical study, improving both patient safety and clinical outcomes.

## Clinical Relevance—

This is a prototype of a continuous monitoring and email-notification system that will allow clinicians to automatically monitor patient states and receive notifications of behavioral changes, thereby improving patient outcomes and increasing the safety of clinical trials, especially those where stimulation may have an unknown effect on the patient while outside of the clinic.
